# Accelerate postoperative management after scoliosis surgery in healthy and impaired children: intravenous opioid therapy versus epidural therapy

**DOI:** 10.1007/s00402-021-03972-3

**Published:** 2021-07-24

**Authors:** Katharina Dinter, Henriette Bretschneider, Stefan Zwingenberger, Alexander Disch, Anne Osmers, Oliver Vicent, Falk Thielemann, Jens Seifert, Peter Bernstein

**Affiliations:** 1 UniversityCenter for Orthopaedic, Trauma and Plastic Surgery , University Comprehensive Spine Center, University Medicine “Carl Gustav Carus” , TU Dresden, Fetscherstrasse 74, 01307 Dresden, Germany; 2 Department of Anesthesiology and Intensive Care Medicine, University Hospital “Carl Gustav Carus”, TU Dresden, Fetscherstrasse 74, 01307 Dresden, Germany; 3 Department of Spine Surgery, AKG Klinik Hohwald GmbH, Hospital for Orthopaedics and Rheumatology, Hohwaldstraße 40, 01844 Neustadt in Sachsen, Germany

**Keywords:** Epidural analgesia, Scoliosis surgery, Pain, Recovery

## Abstract

**Purpose:**

Postoperative pain is a major concern following scoliosis surgery. CEA (continuous epidural analgesia) is established in postoperative pain therapy as well as intravenous patient-controlled analgesia (IV-PCA). The purpose of this study was to compare the clinical outcomes of both methods.

**Methods:**

We retrospectively studied 175 children between 8 and 18 years who were subject to posterior scoliosis correction and fusion. Two main cohorts were formed: CEA with local anesthetic and opioids, and IV-PCA with opioids. Both groups further comprised two sub-cohorts: those who were mentally and/or physically healthy (H; *n* = 93 vs. *n* = 30) and those who were impaired (I; *n* = 26 vs. *n* = 26). The outcome parameters were the demand for pain medication, parameters of mobilization, and the presence of adverse reactions.

**Results:**

Healthy children who received CEA started mobilization 1 day earlier than children with IV-PCA (*p* = 0.002). First postsurgical defecation was seen earlier in all children who received CEA in both groups (H; Day 4 vs. Day 5, *p* = 0.011, I; Day 3 vs. Day 5, *p* = 0.044). Healthy children who received CEA were discharged from hospital 4 days earlier than their IV-PCA counterparts (*p* < 0.001). No statistically significant difference in postoperative nausea nor in vomiting was identified between groups. Transient neurological irritations were seen in 9.7% of the patients in the CEA group.

**Conclusions:**

CEA provides appropriate pain management after scoliosis surgery, regardless of the patient’s mental status. It allows earlier postoperative defecation for all patients , as well as shorter hospitalization and an earlier mobilization for healthy patients.

## Introduction

Tridimensional deformity of the spine, also known as scoliosis, can have a huge impact on the patients quality of life. Surgical correction of idiopathic scoliosis is considered for curves above 45–50° [1]. Due to the large wound area and the spinal correction, patients suffer from severe pain after scoliosis surgery. The treatment of this postoperative pain remains one of the major challenges in scoliosis surgery.

Postsurgery pain can be a very distressing feature of scoliosis surgery, and it can increase postoperative morbidity, complication rates, and length of hospitalization (LOH) [2]. Therefore, pain management takes a central role in postoperative patient treatment and needs to be optimized continuously. For many years, opioids administered via intravenous patient-controlled analgesia (IV-PCA) remained the gold standard for postoperative pain management after scoliosis surgery.

Epidural application of pain medication, such as continuous epidural analgesia (CEA), emerges as an efficient postoperative pain management method. It has been shown that epidural catheters can be used safely and are an efficient means of pain management [3] in patients suffering from Adolescent Idiopathic Scoliosis (AIS) [4], and in children with neuromuscular scoliosis (NMS) [5]. If the catheter is placed correctly, it can provide good analgesia after posterior spinal fusion [6]. For this reason, it is now a widely used method.

In scoliosis surgery, placing an epidural catheter after posterior correction is straightforward: The surgical field is prepared already and the placement itself does not require the spinal surgeon to have completed additional training. Thus, epidural catheters were used across multiple centers.

However, whether CEA is superior to intravenous opioid therapy via IV-PC remains a matter of debate as contradictory data can be found in the literature.

Despite the proven advantages in other applications, some concerns remain: catheters might be an additional source of infection by channeling the way for bacteria during their application; and, since they can only be applied in an inpatient setting, they can further prolong the hospital stay. This trial aims to answer the question of whether CEA provides better outcomes than IV-PCA in healthy and impaired children after Posterior Spinal Instrumented Fusion (PSIF).

## Methods

This study is a retrospective cohort analysis between 2004 and 2016, and has been approved by the institutional review board (#EK111032017). For pain management, IV-PCA was used until 2008 and switched to CEA in 2009.

**Fig. 1:**
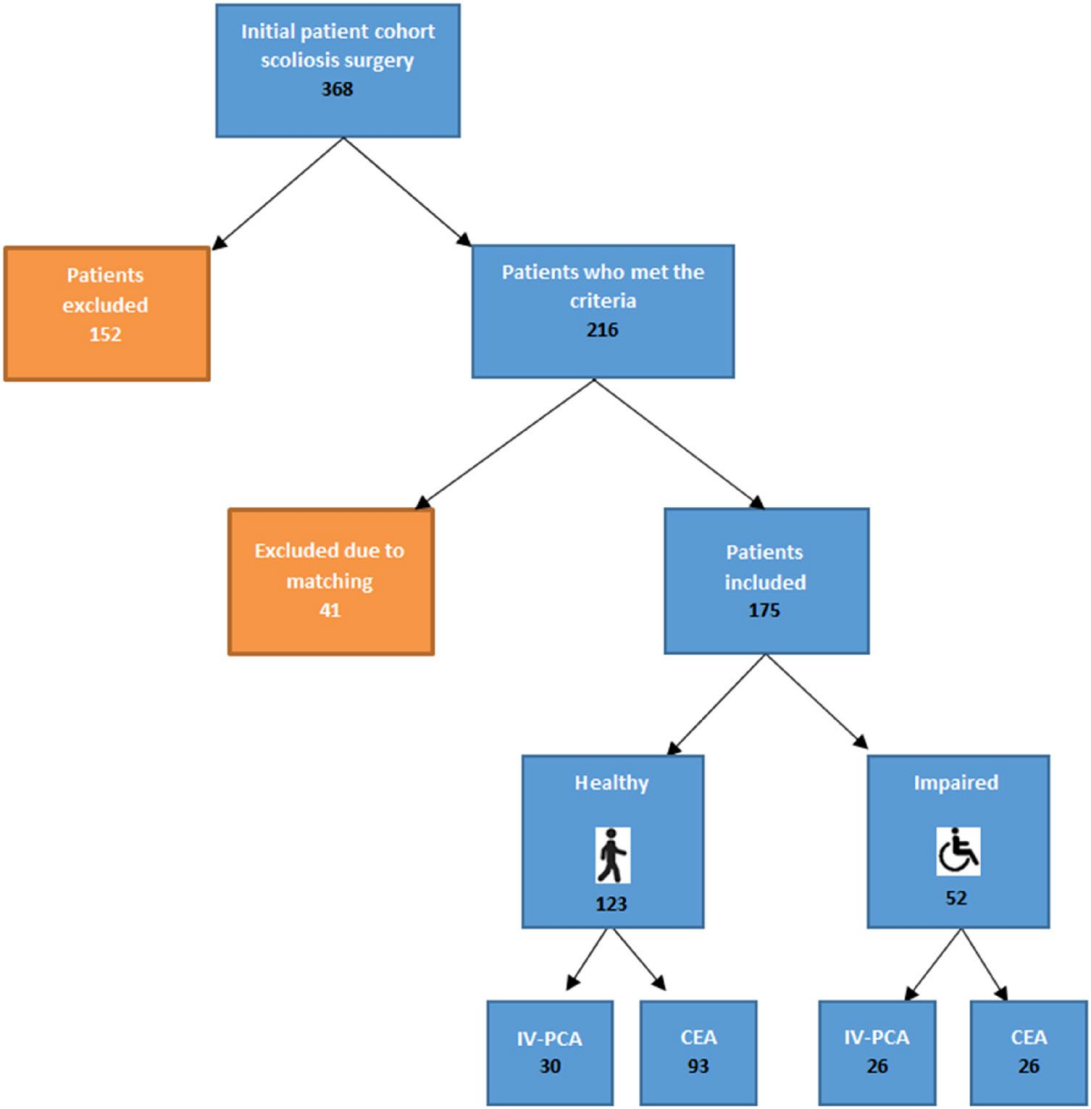
Selection of patients and division into the groups depicted as flowchart. Initially, 368 patients were enrolled. After exclusion of 193 patients due to several reasons, 175 were included in the study

The patients were selected according to the following protocol (Fig. 1).

All patients were subject to PSIF using multilevel pedicle screw constructs by various suppliers. Patients older than 18 years or who had modified procedures (e.g., additional thoracoplasty, anterior correction, and hemivertebra resection) were excluded. All patients were operated by JS, FT, or PB. Children received either IV-PCA or CEA for postoperative pain management. Data were provided through the patient records (paper or digital).

Patients were divided into two groups on the basis of their mental and/or physical status into healthy and impaired. In this article, healthy children (**H**) are depicted with the symbol of a walking person (), whereas the mentally and/or physically impaired children (**I**) are depicted by a wheelchair (). An arrangement into different groups was necessary, as impaired children mostly mobilize via wheelchair, whereas healthy children mobilize independently. In addition, impaired children are often not able to communicate pain adequately or administer pain medication independently, so these groups had to be analyzed separately.

Furthermore, the groups were classified according to pain management (IV-PCA, CEA), so that four groups were formed. Table 1 shows the grouping.

**Table 1:** Division into four groups was performed due to the mental and/or physical status and the pain-management system (IV-PCA, CEA)

	
$\frac{\text{Group 1}}{{IV-PCA}}$	$\frac{\text{Group 2}}{{CEA}}$	$\frac{\text{Group 3}}{{IV-PCA}}$	$\frac{\text{Group 4}}{{CEA}}$

Table 2 presents the etiologies of the scoliotic deformities.

**Table 2:** Etiologies of the scoliotic deformities in the four groups

	
$\frac{\text{Group 1}}{{IV-PCA}}$	$\frac{\text{Group 2}}{{CEA}}$	$\frac{\text{Group 3}}{{IV-PCA}}$	$\frac{\text{Group 4}}{{CEA}}$
Idiopathic (29)	Idiopathic (89)	Duchenne muscular dystrophy (5)	Duchenne muscular dystrophy (7)
Neurofibromatosis (1)	Marfan syndrome (1)	Cerebral palsy (CP) (11)	Cerebral palsy (CP) (12)
	Congenital scoliosis (1)	Ganglioglioma (2)	Borjeson−Forssman−Lehmann syndrome (1)
	Scolosis due to scaring (1)	Astrocytoma (1)	Spinal muscular atrophy (SMA) (1)
	Scheuermann's disease (1)	Dandy Walker syndrome (2)	Rette Syndrome (1)
		Freeman−Sheldon syndrome (1)	Down Syndrome (1)
		Myelomeningocele/Hydrocephalus (1)	Pelizaeus–Merzbacher-like disease (1)
		Minicore (Multicore) myopathy (1)	Alpha-dystroglycanopathy (1)
		Unknown myopathy (1)	Smith–Lemli–Optiz syndrome (1)
		Neurofibromatosis (1)	

Thus, from 368 patients, a total of 216 were eligible for analysis, 123 belonging to the healthy ( ) and 93 to the impaired group ( ). Exclusion criteria are listed in Table 3.

**Table 3:** Exclusion criteria: 193 patients were excluded from the trial, due to several reasons

Exclusion criteria	Total number
Pain management other than CEA or IV-PCA	12
Age (> 18 years)	111
Combined surgeries (anterior + posterior correction)	3
Patient records not accessible for research	20
Patient received other surgery than posterior correction	6
Exclusion due to matching	41
	193

To achieve age- and sex-matched cohorts, further 41 patients from the impaired group had to be excluded by matching, resulting in a total number of 175 patients ( : 123, : 52)

**Fig. 2:**
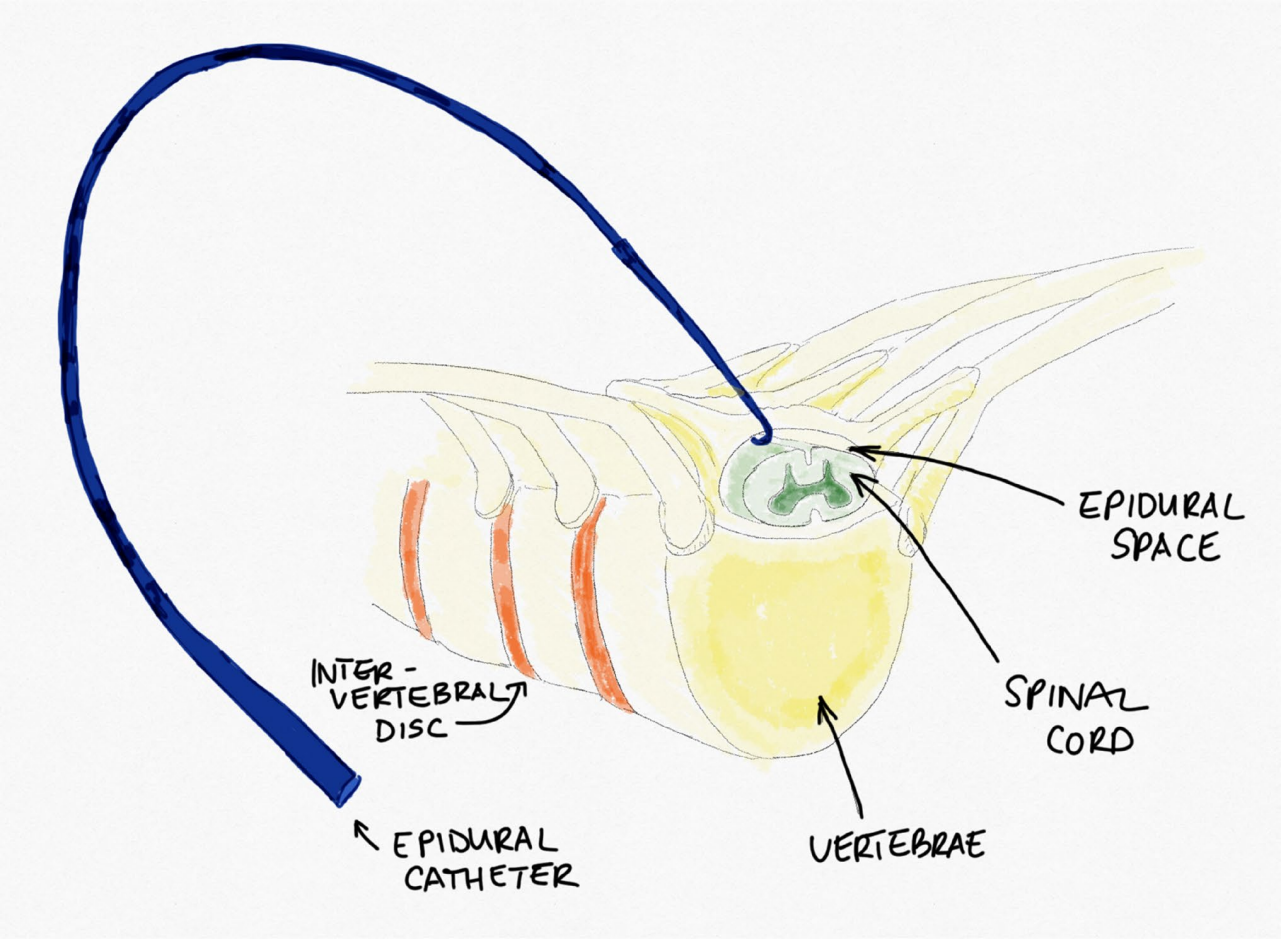
Epidural catheter insertion. After surgical correction, the spinal canal is opened interlaminar in the midline. The catheter is inserted into the epidural space and gently pushed cranially, so that the tip of the epidural catheter is located 5–8 cm above the insertion site

Technically, epidural catheters were placed through a 3–4 mm-wide interlaminar midline opening to the epidural space, thus allowing free passage and preventing leakage of anesthetics. The catheter was introduced at the level of the thoracic apex (e.g., between Th8 and Th9) and gently pushed cranially to secure for 5–8 cm epidural location (Fig. 2).

To compare the efficiency of the pain-medication regimes, the following parameters were studied: (Table 4)

**Table 4:** Parameters to objectify and measure the efficiency of the pain-management systems

Outcome	Pain	Adverse reactions	Complications
First defecation First mobilization Length of hospitalization (LOH) Stay on Intensive Care Unit (ICU)	Number of times, the patient operated the pump Additional demand for Opioids Additional demand for Non-Opioids	Nausea Vomiting Hypotension Neurological irritations	Complications

They were divided into four groups: outcome, pain, adverse reactions, and complications

First defecation and discharge from ICU were calculated as postoperative days and the length of hospital stay was counted in full days. First mobilization was defined as “standing in front of the bed” for healthy children and “transfer into the wheelchair” for impaired children.

Assessment of pain was expressed through the number of times the patients administered drugs via the catheter or IV-PCA or demanded additional pain medication (rescue medication), such as opioids and non-opioids. Some of the impaired children were not able to operate the pump or ask for additional pain medication themselves. In these cases, the nursing staff or parents administered the pain medication. Nausea and vomiting were counted when mentioned in the patient notes.

If an adverse reaction resulted in any further operative intervention, intensive medical treatment, or prolonged hospital stay, it was defined as a complication.

Postoperative pain therapy via CEA was delivered by a continuous and patient-triggered application of Ropivacaine 0.2% + 0.5 μg/ml Sufentanil through the epidural catheter, the volume being adjusted to patient body weight and level of pain. Patients using IV-PCA for postoperative pain management were able to self-administer weight-adjusted boluses of Piritramide when feeling pain (bolus volume 2–4 ml). In this system, no continuous application was established as the administration of Piritramide was only done through bolus application (volume being adjusted to patient’s body weight).

Weaning off the epidural catheter was started 3–4 days postsurgery by reducing the continuous rate and followed by removal of the catheter on the fifth day. During CEA—and IV-PCA weaning, as well as rescue medication, various drugs were used (Piritramide, Metamizole, and Nonsteroidal anti-inflammatory drugs [NSAID]).

Statistical analysis was conducted using SPSS-statistics software. For significance detection, Chi-squared test, as well as the Mann–Whitney U test, were performed. Differences in means were considered significant when *p* < 0.05. A comparison was explicitly carried out between the application systems within the same mental/physical status groups. Therefore, impaired children were never compared to healthy children.

## Results

There was no statistically significant difference in terms of age, weight, and height between the groups. Age, sex distribution, instrumentation lengths, and duration of surgery of the patient groups are shown in Table 5.

**Table 5:** Age, sex distribution, instrumentation lengths, and duration of surgery of the patient groups in medians and range, as well as Percentage; median ± SD (min/max)

		
	$\frac{\text{Group 1}}{{IV-PCA}}$	$\frac{\text{Group 2}}{{CEA}}$	$\frac{\text{Group 3}}{{IV-PCA}}$	$\frac{\text{Group 4}}{{CEA}}$
Age (years)	15 ± 2 (12–18)	14 ± 2 (10–18)	14 ± 2 (8–18)	14 ± 2 (11–18)
Sex distribution (Female:male)	70%:30%	81%:19%	35%:65%	27%:73%
Instrumentation length (segments)	12 ± 2 (6–14)	12 ± 2 (6–15)	13 ± 2 (7–16)	14 ± 2 (7–16)
Duration of the surgery (Minutes)	179 ± 34 (102–253)	181 ± 54 (98–436)	185 ± 62 (115–401)	183 ± 45 (140–292)

**Fig. 3:**
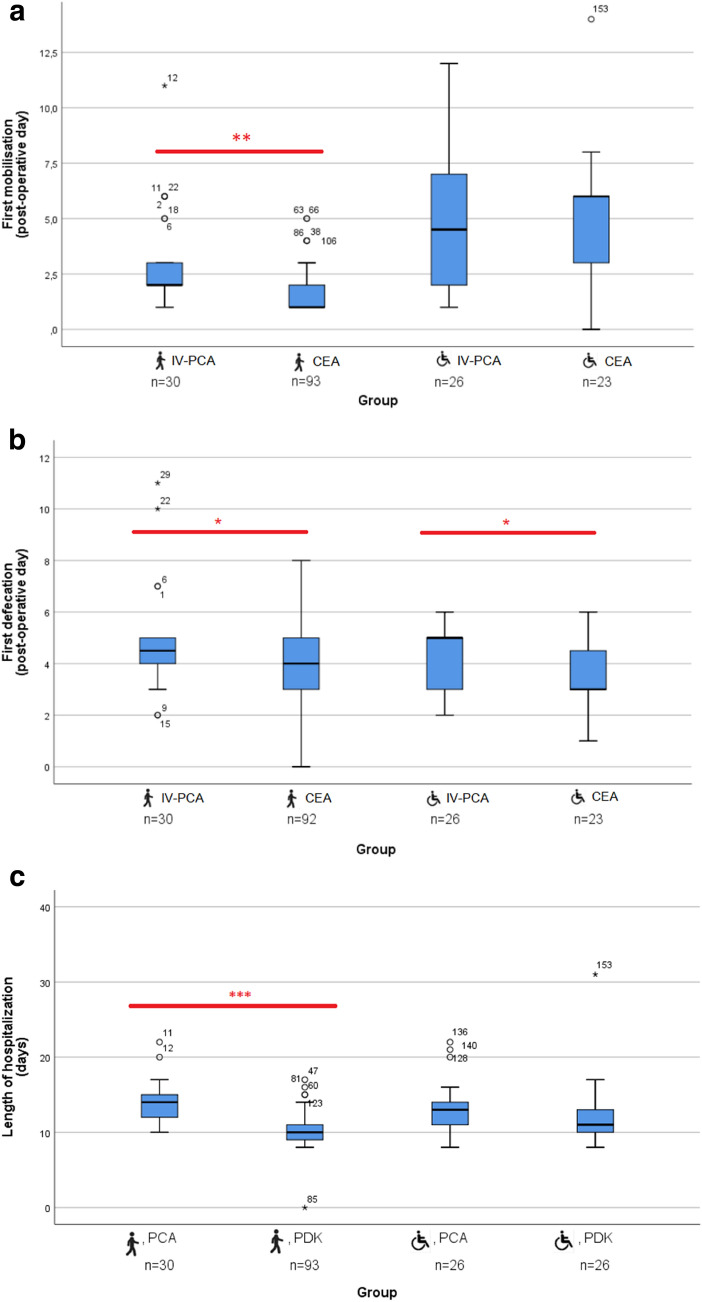
**a** Boxplots of each group depicting first mobilization of healthy ( ) and impaired ( ) children. **p = 0.002. Extreme outliers are marked with an asterisk (*) on the boxplot. Mild outliers are marked with a circle (O) on the boxplot. **b** Boxplots of each group depicting first defecation of healthy ( ) and impaired ( ) children. *p < 0.05. Extreme outliers are marked with an asterisk (*) on the boxplot. Mild outliers are marked with a circle (O) on the boxplot. **c** Boxplots of each group depicting length of hospitalization of healthy ( ) and impaired ( ) children. ***p < 0.001. Extreme outliers are marked with an asterisk (*) on the boxplot. Mild outliers are marked with a circle (O) on the boxplot

Healthy patients who had received epidural anesthetics could be mobilized 1 day earlier than IV-PCA-treated patients (median day 1 vs. 2 postsurgery, *p* = 0.002). However, the same effect could not be reproduced for wheelchair mobilization in impaired children, who remained bed-bound for about 5 days, regardless of postoperative pain therapy (Fig. 3a).

**Fig. 4:**
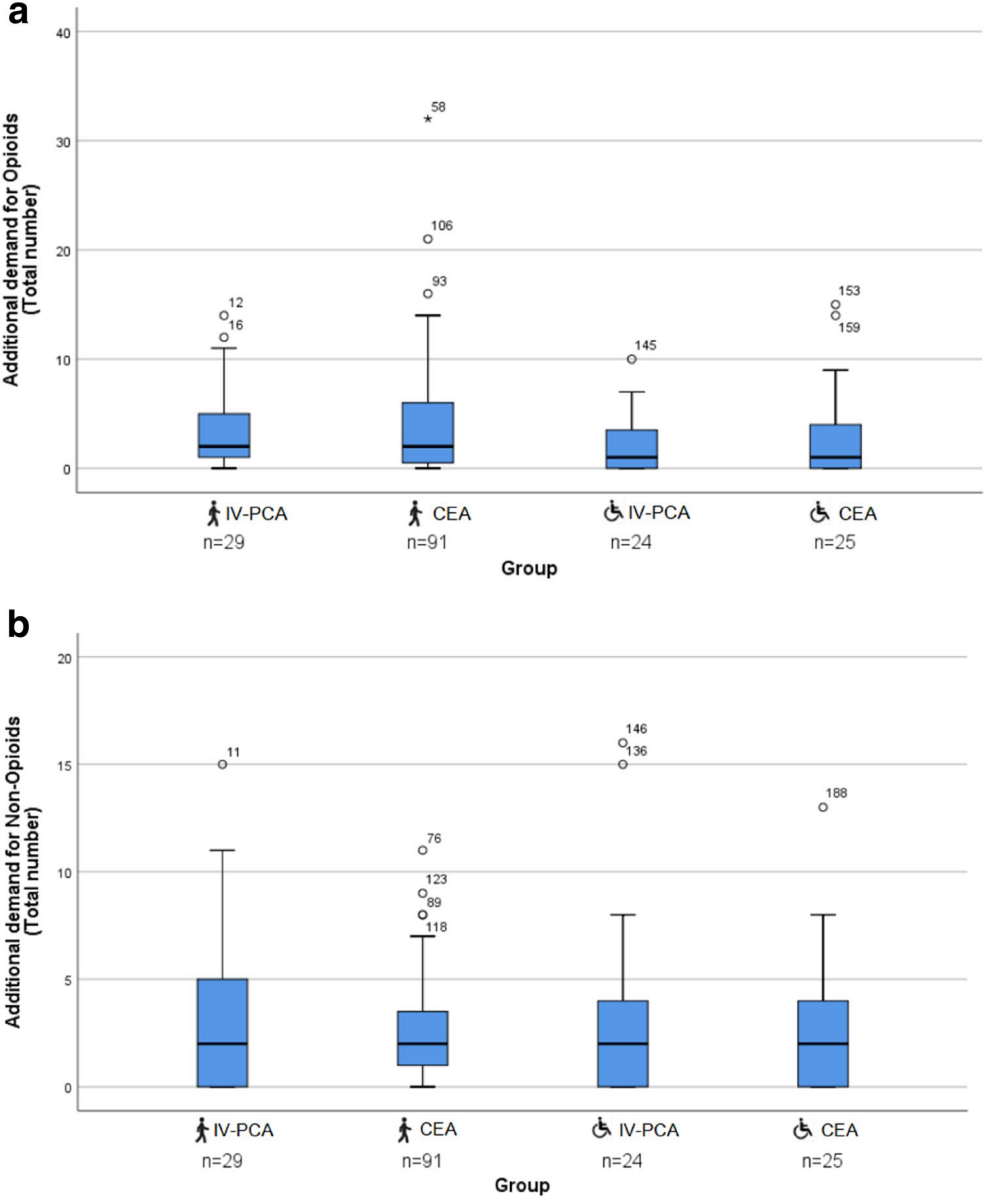
**a** Boxplots of each group depicting additional demand for Opioids (total number) of healthy ( ) and impaired ( ) children. Extreme outliers are marked with an asterisk (*) on the boxplot. Mild outliers are marked with a circle (O) on the boxplot. **b** Boxplots of each group depicting additional demand for non-opioids (total number) of healthy ( ) and impaired ( ) children. Extreme outliers are marked with an asterisk (*) on the boxplot. Mild outliers are marked with a circle (O) on the boxplot

First postsurgical defecation was seen earlier in all patients who received epidural anesthetics, compared to those who received IV-PCA, speeding up bowel movement for 1 day in healthy children (*p* = 0.011) and 2 days in impaired children (*p* = 0.044) (Fig. 3b).

Healthy children who had received CEA were discharged from hospital around 4 days earlier than their IV-PCA-treated counterparts (*p* < 0.001). This effect was not as pronounced in impaired children (Fig. 3c), but a trend towards earlier discharge could be seen as well. LOH was counted in full days.

There was no difference in additional demand for pain medication in healthy and impaired children, regardless of the type of postoperative pain management (Fig. 4a, b).

No differences between the cohorts have been detected in occurrence of postoperative nausea (64%–70% in healthy, *p* = 0.582; 46%–54% in impaired, *p* = 0.579) nor in vomiting (48%–60% in healthy (*p* = 0.269), 34%–42% in impaired, (*p* = 0.569)) or in postoperative hypotension.

There were 9 catheter-associated neurological irritations in group 2 (9.7%) and no irritations in group 4. Sensory disturbances (upper or lower extremities) were seen in 6 cases. In 3 cases, patients described a hypoesthesia (chest, upper extremities, lower extremities), and in 1 case, a paresis was found (upper extremities). The irritations were either treated through a change of therapy (pause of therapy or catheter removal) or it resolved independently, without any change in therapy. In one case, an MRI was performed without any further therapeutic implications. All neurological deficits resolved completely during the hospital stay. No epidural leakage was found postoperatively.

There were 2 severe complications, where a causing by the catheter could not be ruled out completely. One was an early wound infection in the epidural catheter group, which required surgical intervention. The other complication was a vegetative decompensation, presenting as hypotension and respiratory arrest after first commissioning of the epidural catheter.

## Discussion

Due to the large wound area and the spinal correction, patients suffer from intense pain which can be a distressing and disabling side effect of scoliosis surgery. Most scoliosis centers use IV-opioids for postoperative pain management; however, these drugs are associated with considerable side effects [7].

In our center, we settled on using CEA for postoperative analgesia. Technically, the placement of an epidural catheter at the end of a posterior spinal procedure is a straightforward procedure and should ameliorate the postoperative outcome.

In our study, we showed that significant milestones of postoperative recovery, such as early bowel movement and mobilization, can be accelerated. Compared to IV-PCA-treatment, healthy children receiving CEA could be ambulated one day earlier. Both groups, healthy and impaired children, were found to have earlier bowel movements than their IV-PCA counterparts.

Pain is not only a distressing sensation, and it increases the complication rate as well as the LOH [2]. Epidural analgesia was shown to be a safe and effective pain management regime after major spinal surgery in patients suffering from AIS [4], as well as patients with NMS [5]. It is difficult to evaluate postoperative pain therapy by the amount of pain or the need for pain medication. Healthy as well as impaired children have various individual ways to communicate discomfort, depending on a variety of factors, including expectation, education and cognitive capacity, anxiety, and assistance by caregivers. Mentally impaired children were often unable to express pain verbally. This lack of communication constitutes a risk of underestimating pain in this group of children, shown by *Shrader* [8]. He reported that patients with AIS received more than twice the amount of narcotics compared with patients with neuromuscular scoliosis. Unfortunately, there is a lack of standardization in the method of pain assessment across studies in children with mental impairment [9].

For this reason, studies proving the superiority of any pain medication regime in this particular group gain special emphasis*.*

We could not demonstrate the superiority of CEA over IV-PCA in pain management in our study. Yet, it was shown in several other trials for AIS [7, 10, 11] or NMS [5]. However, these results need to be reflected critically, as *Saito's* [5] study cohort of impaired children only consisted of 10 patients when our cohort counted 52. *Gauger* [10] conducted a prospective study, which is concerning the study design, stronger, but only included 38 patients. In comparison, our study contained 123 patients in the non-impaired group. Only *Meng *[11] provided a larger number of participants, when comparing 17 trials (RCT) of major spinal surgery, which compared IV-PCA and CEA. He could show that CEA provides significantly superior analgesia and higher patient satisfaction. Yet, one thing that seems to be clear and logical is that CEA reduces the amount of opioid use [7, 11, 12] and thereby reduces opioid-induced side effects.

It is known that CEA has a positive impact on early bowel movement [13]. Early mobilization and early bowel movement have a positive impact on LOH after PSIF [14] and the overall complication rate in spinal surgeries [15].

We found the first postsurgical defecation earlier in all groups receiving CEA compared to those with opioid analgesics. Similarly, findings were published by *Cassady* [16] whose data indicated that resumption of bowel sounds occurred earlier in patients receiving CEA than in those receiving IV-PCA. *Van Boerum* [9] showed that patients in the epidural group tolerated a full diet earlier (*p* = 0.03) than patients in the IV-PCA group.

Our results clearly show that non-impaired patients who received CEA after posterior spinal fusion in adolescent scoliosis are able to ambulate earlier than patients receiving IV-PCA. These findings are contradictory to other publications [4, 9], that could not find any difference in the time until independent ambulation. Apart from an effective pain-management through CEA (as was shown by other studies [11]), this could also be due to the rising awareness of the importance of early mobilization after major surgery.

In a few cases, installation and use of CEA might be associated with complications, such as hypotension or bronchoconstriction [13]. Neurological irritations, such as numbness or tingling sensations and transient motor blockage, are a well-known concomitant feature of CEA and are described in the literature [4, 7, 10, 12, 17]. In our trial, all neurological irritations resolved during the hospital stay.

We observed one case of vegetative decompensation, presenting as hypotension and respiratory arrest after the first injection via the epidural catheter and transferring the patient into the bed. A similar case is described by *Gauger *[10], who reported one case of hypotension which required epidural discontinuation. The adverse reaction in our study might be caused by a lack of sympathetic reaction to an acute volume distribution.

The occurrence of wound infection caused by the catheter is a rarely described complication in literature. *Cassady* [16] also found one wound infection in his trial, yet stated that there is no significant relationship to the catheter.

Although prone to dysfunction in some patients, epidural catheters are generally safe [3–5] and do not cause longer operation times or more infections.

## Limitations

Measuring postoperative pain remains one of the most important tasks in the surgical field. Since it is still a challenge to measure pain in a prospective manner, it is even more difficult to assess it retrospectively or to assess and compare the total amount of pain medication the patients have received. For this reason, the limitations of our study are of a retrospective nature. We were not able to use generally accepted tools, as the NRS, for it was not used back in 2004. Also, there might be a bias in, for example the LOH or the first time until first mobilization as IV-PCA was used before 2009 and CEA was used afterwards. Before 2009 the awareness of early first mobilization was not as high as it is today and LOH became an economic factor with changes in the German health care system.

A small number of CEA patients needed additional IV-PCA-support to ease the pain. Yet, this transfer was already seen in other trials [9, 10]. *Klatt* [4] described epidural leakage as a reason for inadequate pain control in 10 cases. Besides malposition, this might be a reason for inadequate pain control and the need for additional pain medication. But even though some patients received CEA and opioids, we could show that this group had a better outcome as patients only receiving opioids.

The applicability of CEA is limited in operations where multiple unclosed osteotomies are applied, because the local anesthetics leave the epidural space through the osteotomies. In cases of limited invasive scoliosis surgery (e.g., growing rod insertion), the insertion of the epidural catheter is not favorable.

## Conclusion

This study shows that the epidural catheter can be used as an effective means of postoperative pain management for children with scoliosis, irrespective of mental and non-mental disability. Our results verify that it is more effective than IV-PCA in postoperative pain management after posterior spinal fusion, measured by bowel movement, independent ambulation, and duration of hospital stay in non-impaired children. Regarding impaired children, it is more effective in the normalization of bowel movement, and equally effective in first ambulation, hospitalization, and treatment of postoperative pain.

For non-impaired children, epidural pain therapy clearly accelerates postoperative mobilization, allowing standing up only a few hours after surgery.

Therefore, in our center, the application of CEA after posterior scoliosis correction has become standard treatment.

## References

[1] Klatt JW, Mickelson J, Hung M, Durcan S, Miller C, Smith JT (2013). A randomized prospective evaluation of 3 techniques of postoperative pain management after posterior spinal instrumentation and fusion. Spine.

[2] Meng Y, Jiang H, Zhang C, Zhao J, Wang C, Gao R, Zhou X (2017). A comparison of the postoperative analgesic efficacy between epidural and intravenous analgesia in major spine surgery: a meta-analysis. J Pain Res.

[3] Arms DM, Smith JT, Osteyee J, Gartrell A (1998). Postoperative epidural analgesia for pediatric spine surgery. Orthopedics.

[4] Martin BD, Pestieau SR, Cronin J, Gordish-Dressman H, Thomson K, Oetgen ME (2020). Factors affecting length of stay after posterior spinal fusion for adolescent idiopathic scoliosis. Spine Deformity.

[5] Saito W, Inoue G, Imura T, Takenami T, Ueno M, Nakazawa T, Uchida K, Takahira N, Takaso M (2015). Safety and efficacy of continuous epidural anesthesia following scoliosis surgery in respiratory-impaired neuromuscular children: A Pilot Study. Spine Deformity.

[6] Turner A, Lee J, Mitchell R, Berman J, Edge G, Fennelly M (2003). The efficacy of surgically placed epidural catheters for analgesia after posterior spinal surgery. Anaesthesia.

[7] Blumenthal S, Min K, Nadig M, Borgeat A (2005). Double epidural catheter with ropivacaine versus intravenous morphine: a comparison for postoperative analgesia after scoliosis correction surgery. Anesthesiology.

[8] Shrader MW, Falk MN, Cotugno RS, Jones JS, White GR, Segal LS (2014). Are we undermedicating patients with neuromuscular scoliosis after posterior spinal fusion?. Spine Deformity.

[9] Van Boerum DH, Smith JT, Curtin MJ (2000). A comparison of the effects of patient-controlled analgesia with intravenous opioids versus Epidural analgesia on recovery after surgery for idiopathic scoliosis. Spine.

[10] Gauger VT, Voepel-Lewis TD, Burke CN, Kostrzewa AJ, Caird MS, Wagner DS, Farley FA (2009). Epidural analgesia compared with intravenous analgesia after pediatric posterior spinal fusion. J Pediatric Orthopaedics.

[11] Ostojic K, Paget S, Morrow A (2019). Management of pain in children and adolescents with cerebral palsy: a systematic review. Develop Med Child Neurol.

[12] Blumenthal S, Borgeat A, Nadig M, Min K (2006). Postoperative analgesia after anterior correction of thoracic scoliosis: a prospective randomized study comparing continuous double epidural catheter technique with intravenous morphine. Spine.

[13] Avila-Hernandez AN, Singh P. Epidural Anesthesia. [Updated 2020 Mar 31]. In: StatPearls [Internet]. Treasure Island (FL): StatPearls Publishing; 2020 Jan

[14] Bellaire LL, Bruce RW, Ward LA, Bowman CA, Fletcher ND (2019). Use of an accelerated discharge pathway in patients with severe cerebral palsy undergoing posterior spinal fusion for neuromuscular scoliosis. Spine Deformity.

[15] Burgess LC, Wainwright TW (2019). What is the evidence for early mobilisation in elective spine surgery? A narrative review. Healthcare.

[16] Cassady JF, Lederhaas G, Cancel DD, Cummings RJ, Loveless EA (2000). A randomized comparison of the effects of continuous thoracic epidural analgesia and intravenous patient-controlled analgesia after posterior spinal fusion in adolescents. Reg Anesth Pain Med.

[17] O’Hara JF Jr, Cywinski JB, Tetzlaff JE, Xu M, Gurd AR, Andrish JT (2004) The effect of epidural vs intravenous analgesia for posterior spinal fusion surgery.Pediatric Anesthesia.14(12):1009–15. Doi:10.1111/j.1460-9592.2004.01387.x 10.1111/j.1460-9592.2004.01387.x 15601351

[18] Janicki J, Alman B (2017). Scoliosis: Review of diagnosis and treatment. Paediatrics Child Health.

